# Multicellular Human Gastric Cancer Spheroids Mimic the Glycosylation Phenotype of Gastric Carcinomas

**DOI:** 10.3390/molecules23112815

**Published:** 2018-10-30

**Authors:** Meritxell Balmaña, Stefan Mereiter, Francisca Diniz, Tália Feijão, Cristina C. Barrias, Celso A. Reis

**Affiliations:** 1 i3S-Instituto de Investigação e Inovação em Saúde, Universidade do Porto, 4200-135 Porto, Portugal; mbalmana@ipatimup.pt (M.B.); smereiter@ipatimup.pt (S.M.); fdiniz@ipatimup.pt (F.D.); taliaf@ibmc.up.pt (T.F.); ccbarrias@ineb.up.pt (C.C.B.); 2 IPATIMUP-Institute of Molecular Pathology and Immunology, University of Porto, 4200-135 Porto, Portugal; 3 INEB-Instituto de Engenharia Biomédica, University of Porto, 4200-135 Porto, Portugal; 4 Institute of Biomedical Sciences of Abel Salazar–ICBAS, University of Porto, 4050-313 Porto, Portugal; 5 Medical Faculty, University of Porto, 4200-319 Porto, Portugal

**Keywords:** 3D cell culture, gastric cancer, glycosylation, MUC1, spheroids, swainsonine, tunicamycin

## Abstract

Cellular glycosylation plays a pivotal role in several molecular mechanisms controlling cell–cell recognition, communication, and adhesion. Thus, aberrant glycosylation has a major impact on the acquisition of malignant features in the tumor progression of patients. To mimic these in vivo features, an innovative high-throughput 3D spheroid culture methodology has been developed for gastric cancer cells. The assessment of cancer cell spheroids’ physical characteristics, such as size, morphology and solidity, as well as the impact of glycosylation inhibitors on spheroid formation was performed applying automated image analysis. A detailed evaluation of key glycans and glycoproteins displayed by the gastric cancer spheroids and their counterpart cells cultured under conventional 2D conditions was performed. Our results show that, by applying 3D cell culture approaches, the model cell lines represented the differentiation features observed in the original tumors and the cellular glycocalix underwent striking changes, displaying increased expression of cancer-associated glycan antigens and mucin MUC1, ultimately better simulating the glycosylation phenotype of the gastric tumor.

## 1. Introduction

Gastric cancer is the third leading cause of cancer-related deaths worldwide, and despite the efforts performed to find novel treatments, surgery remains the only curative treatment for localized gastric cancer [1]. However, large numbers of gastric cancer patients are diagnosed when metastases are already present, and then curative attempts are vain. At present, chemotherapy is used as first-line treatment for these patients. Several drugs with higher specificity are currently being developed, but good in vitro models that simulate in vivo physiology are required to assess their efficacy.

The culture of mammalian cells has been classically performed in two-dimensional (2D) setups. The increasing knowledge of tumor biology, in which architecture plays an important role, has led to the development of novel approaches allowing improved simulation of physiological conditions. These systems can mimic the complex structures of different types of tissue in the mammalian body, helping to overcome the limited prediction accuracy of classical 2D-based screening assays [2,3]. Multicellular spheroids methods, where cells cluster together to form unique entities, have been demonstrated to be promising for the screening of novel drugs [4]. Different approaches have been developed for the formation of multicellular tumor spheroids (MCTS) [5]. Some conventional methodologies include plate coating with polyHEMA or the hanging-drop system, both of which are labor-intensive. As an emerging field with a broad range of applications, innovative methodologies are continuously developed, focusing on exploiting automation and high-throughput capacity. In this regard, microfluidic platforms that allow the formation of a thousand MCTS within a chip have been recently reported [6,7].

The cell glycocalyx is known to be of utmost relevance for a large variety of biological processes, such as cell–cell recognition, communication, intercellular adhesion, leukocyte trafficking and innate immunity [8,9]. In this regard, alterations in glycosylation occur in different diseases including cancer, playing a pivotal role in malignant transformation and tumor progression [9,10]. For this reason, understanding the role of glycosylation in the formation and maturation of multicellular spheroids is crucial in order to use these models for biological and screening studies. In this work, we optimized three-dimensional (3D) spheroid culture conditions for four commonly used gastric cancer cell lines (AGS, MKN45, MKN74, and NCI-N87) with two distinct methods, and characterized in depth the spheroid glycosylation profile as opposed to 2D-cultured cells. In addition, we analyzed the effect of glycosylation inhibition in spheroid development between the four cell line models. To the best of our knowledge, this is the first work addressing the glycosylation displayed by gastric cancer cell spheroids.

## 2. Results

### 2.1. Gastric Cancer Spheroids Morphological Features and Maturation

**Figure 1:**
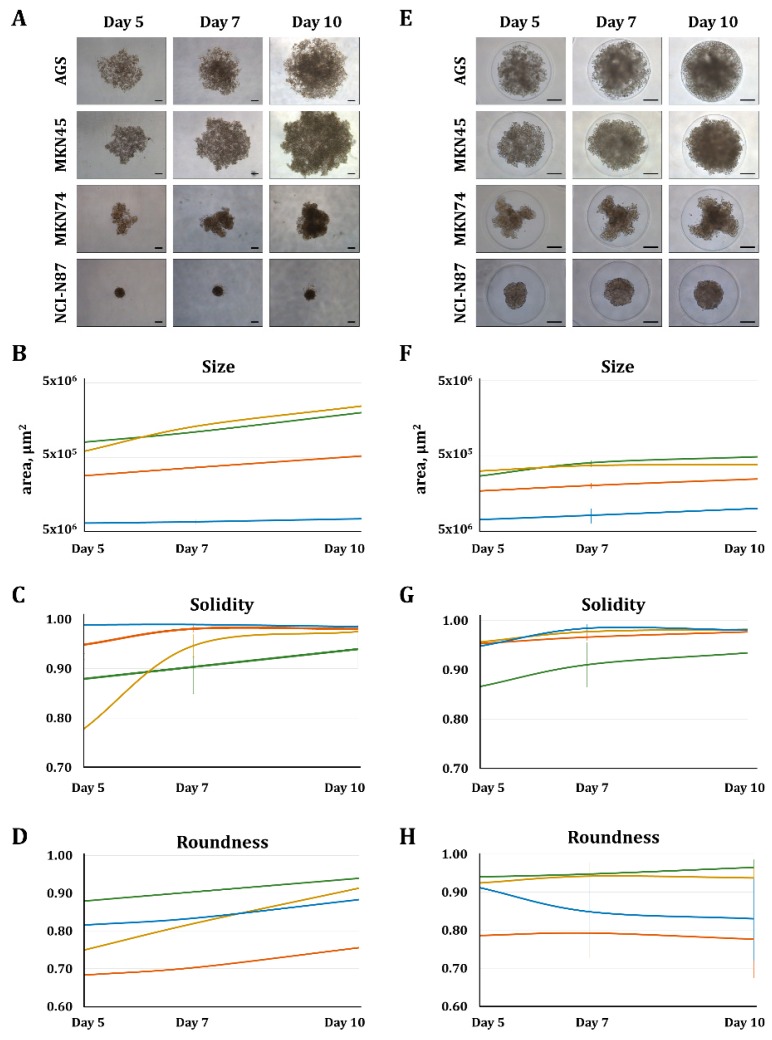
Comparison between two standardized methodologies (ULA and 3D Petri Dish^®^) for generation, imaging, and automatized analysis of gastric multicellular tumor spheroids (MCTS). (**A**) Representative images of gastric MCTS and the results of automated image analysis for (**B**) size, (**C**) solidity, and (**D**) roundness, generated using ULA 96-well round-bottomed plates. (**E**) Representative images of MCTS and the corresponding results of automated image analysis for (**F**) size, (**G**) solidity, and (**H**) roundness, generated with the 3D Petri Dish^®^ technology (MICROTISSUES^®^). Values are means ± SD of at least *n* = 3 (ULA) and *n* = 9 (3D Petri Dish^®^). For each condition, three independent experiments were performed. Scale bar represents 200 µm.

Different amounts of the four gastric cancer cell lines, AGS, MKN45, MKN74, and NCI-N87, were seeded to assess the number of cells required for MCTS formation. It is considered that spheroids with a diameter between 200 and 500 µm develop decreasing gradients of oxygen, nutrients, and catabolites toward the innermost cells; therefore, mimicking tumor physiology [11]. Previous studies have shown that, after four days, isolated cells can group and form MCTS [4]. For an objective evaluation of MCTS features, namely, size, compactness, and roundness, an automated image-analysis system was set up (Figure S1). This analysis allowed us to differentiate between loosely formed cell aggregates and different qualities of MCTS. In this regard, AGS, a cell line lacking functional cell–cell adhesion molecule E-cadherin [12,13] formed the loosest spheroids as shown by their large size and low compactness in a time-point- and cell-number-independent manner. MKN45, a cell line known to form homophilic cell–cell connections, formed loosely packed MCTS, as indicated by the slightly smaller size and higher density than AGS aggregates. NCI-N87 formed the tightest MCTS of the evaluated cell lines, as shown by the small size and 100% compactness, even with low cell numbers. Interestingly, MKN74 also formed small and compact MCTS, but this cell line’s MCTS showed low roundness values. Low roundness indicates a high degree of differentiation and organization when these cells grow as spheroids, a feature that cannot be observed when cells are grown in 2D (Figure 1, Figure S2).

Our data show that the two systems used for MCTS formation, ultralow attachment (ULA) and 3D Petri Dish^®^ resulted in gastric MCTS displaying similar roundness values, but smaller and higher compact MCTS when using the 3D Petri Dish^®^ approach (Figure 1). These results are consistent with the fact that the maximal diameter of the wells created with the 3D Petri Dish^®^ system was 800 μm, about 500,000 μm^2^ of area. In this regard, the 3D Petri Dish^®^ is not only a high-throughput system, but also helps the formation of spheroids by forcing the cells to interact in a limited space.

### 2.2. Glycosylation Affects Multicellular Spheroid Growth

**Figure 2:**
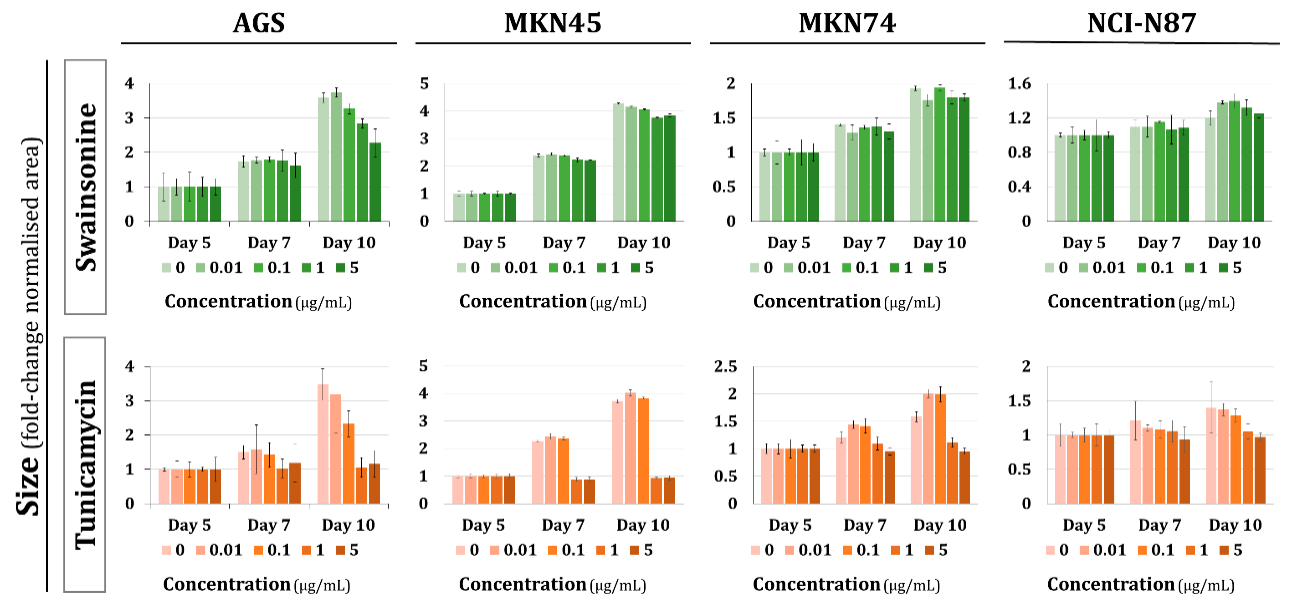
Glycosylation interference analysis for gastric spheroid formation. Multicellular tumor spheroids were grown for five days and treated with different concentrations of glycosylation inhibitor swainsonine or tunicamycin at different time points. Values represent variation in size at each time point in comparison to the value at day 5. Values are means ± SD of at least *n* = 3 spheroids. For each condition, two independent experiments were performed.

The role of glycosylation in MCTS generation was assessed by subjecting the gastric MCTS to two different inhibitors of glycosylation using the ULA plates: tunicamycin, which blocks the synthesis of *N*-glycans; and swainsonine, which prevents the formation of complex *N*-glycans. The results disclosed that MCTS growth is restrained when altering glycosylation (Figure 2). At higher concentrations of tunicamycin (1–5 μg/mL), where the *N*-glycosylation of the proteins at the endoplasmic reticulum is precluded, strong reduction in size was observed in the four cell lines. On the other hand, the effect of swainsonine, a milder inhibitor of glycosylation, as well as the treatment with low concentrations of tunicamycin (0.01–0.1 μg/mL) only showed to affect the growth of the AGS MCTS. Altogether supporting a protective effect of the cells grown in 3D. Since AGS cells do not form cell–cell contacts and display the lowest compactness, this protective effect is lost in the AGS cells (Figure 2). In order to address cell toxicity, we evaluated cell proliferation and the apoptotic status of the treated spheroids. Our results showed that, by treating loose spheroids with swainsonine, no effect was observed on either cell viability or apoptosis. For treatment with tunicamycin, cell proliferation was abrogated and cell death was revealed. On the other hand, compact spheroids showed, for both inhibitors, similar proliferative and apoptotic status as the control. Altogether, these results corroborate the protective effect attributed to strong cell–cell contacts observed in compact MCTS (Figure S3).

### 2.3. Glycosylation Profile in Gastric Multicellular Tumor Spheroids Differs from Cells Cultured in a Monolayer

**Figure 3:**
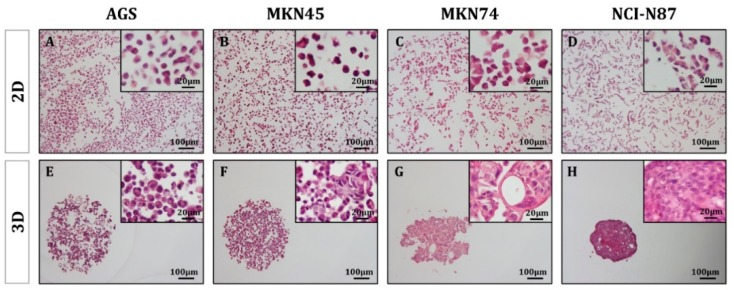
Gastric multicellular tumor spheroid histology. Hematoxylin and eosin staining of representative histological sections of gastric cancer cell lines grown in 2D (**A**–**D**) or 3D conditions (**E**–**H**).

The gastric MCTS were subjected to detailed analysis using a panel of lectins and glycan-directed antibodies to analyze the glycosylation MCTS profile and compare it with the counterpart cells grown under conventional 2D cell culture systems. First, hematoxylin and eosin staining of gastric MCTS showed different histological morphologies (Figure 3). In agreement with the previous results, the AGS were the least compact aggregates (Figure 3E). Similarly, the MKN45 cell line formed loose spheroids, although cell–cell contact points were observed (Figure 3F). On the other hand, both the MKN74 and NCI-N87 cell lines displayed a high degree of cell–cell adhesion and interaction (Figure 3G,H). Special mention should be given to the higher degree of complexity of the MKN74 leading to the formation of well-differentiated glandular-like tissue structures.

**Figure 4:**
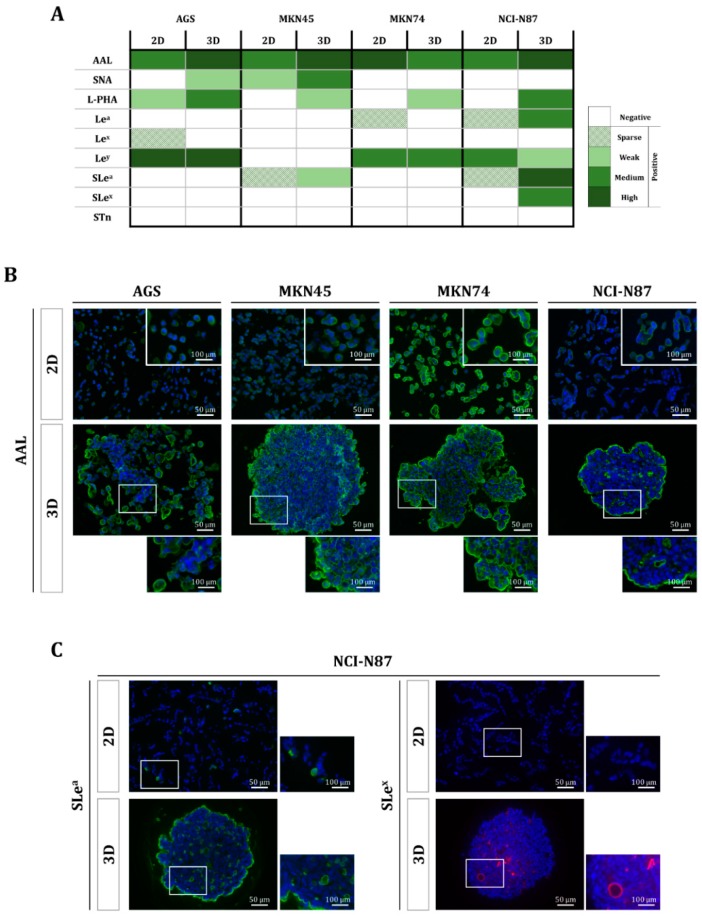
Glycan profiling of the gastric multicellular tumor spheroids. (**A**) Immunofluorescent staining of a panel of lectins and antibodies for glycosylation characterization of gastric cancer cell lines grown in 2D or 3D cell culture conditions. (**B**) *Aleuria aurantia* lectin staining, detecting fucosylation, is shown as a representative example for the four cell lines. (**C**) Differential expression of antigens sialyl Lewis A (SLe^a^) and sialyl Lewis X (SLe^x^) for the NCI-N87 cell line.

The analysis of the glycosylation profile of the four cell lines revealed specific differences when cells were cultured in 3D in comparison with cells grown in monolayer. The gastric MCTS generated with the AGS and MKN45 cell lines, displaying a lower degree of compactness, showed a similar glycosylation pattern as the cells grown in monolayer. On the other hand, MKN74 and NCI-N87, which engage in extensive cell–cell interaction, disclosed a different pattern of staining, with more reactivity with lectins, and antibodies detecting glycans at the outer surface of the MCTS and at the apical membrane of the cancer cells in the glandular-like structures of the MCTS (Figure 4). Different glycosylation features were particularly marked for the expression of sialyl-Lewis A (SLe^a^) and sialyl-Lewis X (SLe^x^) in NCI-N87 when comparing the 3D MCTS to their 2D counterparts (Figure 4C). Additionally, an overall increase of branched structures was observed in all gastric MCTS models (Figure 4A).

### 2.4. Gastric Multicellular Tumor Spheroids Better Resemble Gastric Tumor Tissue

**Figure 5:**
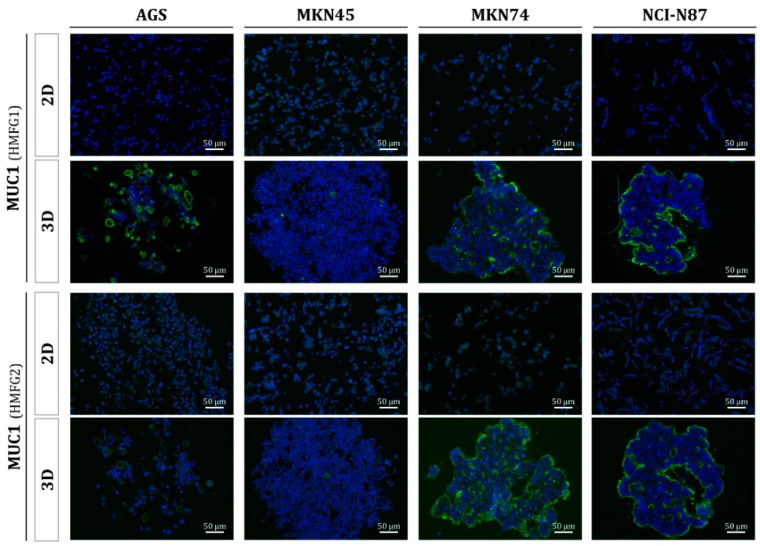
Mucin 1 (MUC1) expression in human gastric multicellular tumor spheroids. Immunofluorescent labelling of the MUC1 expression in gastric cancer cell lines grown in 2D or 3D cell culture methods using two different monoclonal antibodies (HMFG1 and HMFG2).

The organized arrangement of cells into specific multicellular structures has been proven as critical for the functional differentiation of cells. In this work, we tested whether gastric cells grown in 3D were capable of producing mucins, which is a characteristic feature of gastrointestinal epithelial cells [14,15]. As shown in Figure 5, gastric cancer cells grown in monolayer do not express mucins, but gastric MCTS showed expression of the MUC1 mucin, as detected by two different monoclonal antibodies. The expression of MUC1 in the MKN74 and NCI-N87 gastric spheroids is located at the formed glandular-like structures, resembling the phenotype observed in differentiated gastric-cancer tissue [16,17]. 

## 3. Discussion

In the past decades, great efforts have been made to develop cellular model systems that are capable of recapitulating normal and pathological tissue architectures in order to allow studies on normal development and disease mechanisms. Cellular processes are highly influenced by architectural and microenvironmental contexts. Hence, 3D culture models provide invaluable tools to address fundamental biological questions reproducing biologically relevant conditions [2]. 

The improved suitability of cancer spheroids has been highlighted by reports that showed increased resistance of 3D-cultured cancer cells to different antitumoral agents when compared to cells grown in monolayer, better predicting the outcome of clinical trials [2,18]. In addition, tumors grown as 3D multicellular spheroids acquire and sustain a multidrug-resistant phenotype in response to acute drug treatments [19]. Altogether, these models have been shown to be superior over conventional cell cultures in predicting drug efficiencies. In addition, 3D models show improved adhesion features [20,21], and secrete extracellular matrix proteins [22,23] implying that tissue organization, cell adhesion, and the extracellular matrix may synergistically generate apoptosis resistance in metastatic tumors [24]. In this work, we evaluated the capacity of different gastric cancer cell lines to form 3D multicellular aggregates, develop complex cellular structures such as glands, and to express glycans and mucins, which are major components expressed by gastric epithelial cells [15]. 

Different approaches have been developed to culture cells in a 3D context, which can mainly be divided into two classes, scaffold-based and scaffold-free systems [25,26]. In this work, we compared two scaffold-free systems that allowed the multiplexed evaluation of gastric cancer cell spheroids. ULA plates are a rapid method to generate multicellular aggregates by preventing the adhesion of cells to a plastic surface, enforcing the formation of cell–cell interaction. In ULA plates, cells grow in an unconfined area allowing the generation of spheroids with virtually no space restrictions. The fact that there is precisely one spheroid per well also facilitates the development of automated systems for evaluation, including automated plate readers. In contrast, the 3D Petri Dish^®^ approach consists of a more labor-intensive method, requiring an agarose-mold preparation step that assists in the generation of spheroids by driving cell interaction in a limited space. Remarkably, the diversity of available form shapes and sizes enables a more versatile approach for a tailored experimental design. Importantly, this multiplexed method allows for further processing by paraffin embedding of the molds containing the spheroids for histological characterizations. In the present study, we combined the advantages of both methods to evaluate molecular markers as well as to characterize spheroid morphological features. For the latter, we programmed a plug-in into open-access FIJI software to analyze the MCTS in both an objective and automated way. The newly developed image system allowed us to determine not only the size of the spheroids but also to analyze other features, such as the degree of compactness and the roundness of the MCTS. In particular, for the study with glycosylation inhibitors, we selected the ULA plates for their easy automation but, more importantly, due to the possibility that the spheroids would grow in a nonconfined environment, where we could track gastric MCTS growth or regression. Our results using *N*-glycosylation inhibitors support the relevance of glycans in the formation of gastric MCTS; however, further studies addressing *O*-glycosylation are warranted for a complete understanding of the role of the glycome in MCTS.

In this study, the comprehensive evaluation of glycosylation features in these models also generated important glycophenotype information. To prevent artefact results due to different sample processing, the four gastric cancer cell lines, both in 2D- and 3D-culture conditions, were paraffin-embedded following the same procedure. The glycophenotype characterization disclosed a trend of enhanced glycan expression in the 3D MCTS. Among the analyzed glycan determinants, the SLe^a^ and SLe^x^ antigens were demonstrated to be highly expressed in many malignant cancers, and their expression is associated with poor prognosis [9,27,28]. These epitopes are well-known ligands for selectins, being key players in metastasis development [29,30]. Our results showing an increase in tumor-associated carbohydrate antigens, such as SLe^a^ and SLe^x^, can be due to better cell differentiation in a specific orientation, reflecting the different stimuli received by the peripheral cells when compared with the cells in the innermost core region. Our results show that this effect is directly related to the degree of compactness of the MCTS. Moreover, the formation of MCTS allows for more differentiated cancer cell lines to develop a glandular-like structure, whose apical membranes display particularly enriched glycan epitopes and mucins. In order to decipher the specific roles of the differential glycosylation pattern displayed by the gastric MCTS, functional studies are required. The expression of mucins is not only relevant in order to recapitulate physiological conditions, but also has an impact on cell mechanical and rheological properties [31]. In agreement with our results, it has recently been reported that ovarian cancer cell lines increase MUC1 expression when cultured in 3D conditions [32]. Gastric mucosa expresses different mucins that can experience aberrant expression of the mucin core protein, as well as de novo expression of glycan structures during malignant transformation [14,15,33,34], positioning mucins as promising targets in gastric cancer management. In this regard, over the last three decades more than 200 clinical studies have been conducted evaluating mucins as a potential tool for prognosis and therapeutic purposes in a wide range of cancers [35,36,37,38]. Thus, the development of models reproducing the main biological properties of epithelial surfaces in the stomach lumen is of utmost interest and relevance.

In addition, apart from demonstrating changes in glycosylation when cells are cultured with 3D methodologies, our results evidenced that, in contrast to 2D cultures, the four gastric cancer cell lines grown in 3D cultures remarkably displayed differentiation features that were observed in the tumors of origin more than 25 years ago [39,40,41]. In this regard, MKN45 was isolated from a poorly differentiated adenocarcinoma [39], and MKN74 and NCI-N87 from moderately and well-differentiated adenocarcinomas of the intestinal subtype, respectively [39,41]. The AGS model, on the other hand, was isolated from the diffuse subset of a mixed-type gastric carcinoma and thus resembled in 3D culture typical diffuse-type behavior, lacking cell–cell adhesion features [40]. Our findings agree with previously reported studies detailing that gastric MCTS recapitulate the growth pattern and differentiation phenotype of human gastric carcinomas [42]. 

Overall, the present work has achieved, for the first time, a comprehensive evaluation of the glycosylation features of gastric cancer cell lines grown in 3D, disclosing the capacity of the presented approaches and developed models to better recapitulate the in vivo tumor features as compared to conventional methods. The advances in 3D cell culture techniques could bridge the gap between 2D studies and in vivo animal models, providing researchers with more accurate in vitro models that would hasten cancer research and the development of more effective treatments. 

## 4. Materials and Methods

### 4.1. Cell Culture

Four human gastric cancer cell lines (AGS, MKN45, MKN74, and NCI-N87) were used in this study. The AGS and NCI-N87 cell lines were obtained from the American Type Culture Collection (ATCC, Manassas, VA, USA), and the MKN45 and MKN74 cell lines were obtained from the Japanese Cancer Research Bank (Tsukuba, Japan). Cell line identity was authenticated by standard short tandem-repeat-based DNA profiling (STR). All cell lines were grown in a monolayer culture and maintained at 37 °C in an atmosphere of 5% CO_2_, in RPMI 1640 GlutaMAX (Gibco, Thermo-Fisher Scientific, Waltham, MA, USA) supplemented with 10% heat-inactivated fetal bovine serum (Biowest, Riverside, MO, USA). Cultured cell lines were routinely tested for mycoplasma contamination by PCR amplification for mycoplasma pulmonis UAB CTIP, mycoplasma penetrans HF-2, and mycoplasma synoviae 53.

### 4.2. Generation of Gastric Multicellular Tumor Spheroids

MCTS formation was conducted by two different approaches. On the one hand, a different number of cells were either plated in ULA plates (Corning, Thermo-Fisher Scientific) or in 3D Petri Dish^®^ (MICROTISSUES^®^ technology, MicroTissues Inc., Sigma-Aldrich, St. Louis, MO, USA) following the protocol described in Reference [23]. Briefly, 12-series agarose micromolds were prepared using 2% agarose in 0.9% of NaCl. The micromolds were placed in 12-well plates and equilibrated with RPMI 10%FBS before cell seeding. Cell suspension of the desired cell concentration was seeded in the corresponding micromold. A 30 min incubation period was used for cells to settle into the micromolds, and additional medium was finally added to the wells. Spheroid formation, both in the ULA plates and 3D Petri Dish^®^, was monitored with inverted cell culture microscope Leica DMi1 (Leica Microsystems, Wetzlar, Germany).

### 4.3. Spheroid Treatment

The cytotoxic activity of the glycosylation inhibitors tunicamycin and swainsonine (both from Sigma-Aldrich) was followed by monitoring spheroid growth. Aliquots of 1000 AGS cells/well, 250 MKN45 cells/well, 1000 MKN74 cells/well, and 1000 NCI-N87 cells/well were seeded in the ULA plates. After 5 days, when the gastric MCTS were formed, cells were treated with the inhibitors diluted in the cell culture medium at concentrations ranging from 0.01 to 5 μg/mL. Control conditions containing the corresponding amount of DMSO were performed. At day 7, equivalent to 48 h of treatment, microscope images were acquired, and the gastric MCTS were again treated for 72 h (day 10). 

### 4.4. Fluorescent Cell Staining

Cells grown in monolayer were detached with a nonenzymatic cell dissociation reagent (Versene solution, Gibco, Thermo-Fisher Scientific), washed twice with PBS, and fixed with 4% *w*/*v* paraformaldehyde (PFA) for 30 min at RT. Then, cells were resuspended in Richard-Allan Scientific™ HistoGel™ Specimen Processing Gel (Thermo-Fisher Scientific) and paraffin-embedded. At day 5, after seeding the cells in the 3D Petri Dish^®^, the medium was replaced. At day 7, the gastric MCTS were fixed with 4% *w*/*v* PFA for 30 min at RT. Fixed samples were paraffin-embedded. The 2D and 3D blocks containing the four gastric cancer cells were cut into 5 μm sections. Slides were deparaffinised in xylene and rehydrated in sequentially decreasing ethanol concentrations prior to hematoxylin and eosin or immunofluorescence staining. In brief, for the immunofluorescence with antibodies, slides were blocked with nonimmune goat serum in 5% PBS and incubated overnight with the corresponding dilution of the primary antihuman antibody. Slides were then washed with PBS and incubated with the secondary antibodies (antimouse IgG Alexa Fluor^®^-488, antimouse IgM Alexa Fluor^®^-594 or antirabbit Alexa Fluor^®^-488) for 1 h at RT. For fluorescent lectin staining (all from Vector Laboratories, Burlingame, CA, USA) slides were blocked with PBS, 10% BSA, followed by lectin diluted in PBS for 2 h at RT and by FITC-conjugated streptavidin diluted 1/1000 in PBS incubations. Nuclei were counterstained with DAPI and samples were mounted with VectaShield (Vector Laboratories). Sections were imaged with Zeiss Axio Imager Z1 microscope (Carl Zeiss, Oberkochen, Germany) equipped with an AxioCam MR ver.3.0. 

Fluorescence intensity was scored as weak, medium, or high. For this, images of each antigen were acquired applying the same camera settings between 2D and 3D. Samples were scored as sparse whenever they showed less than 10% of positive cells. Evaluation of fluorescence intensity was performed by two independent specialists.

The antibodies used were: Lewis A (Le^a^, CA3F4 [43], dilution 1/5); Lewis X (Le^x^, SH1 [44], dilution 1/5); Lewis Y (Le^y^, AH6 [45], dilution 1/5); Sialyl-Lewis A (SLe^a^, CA19-9, Santa Cruz Biotechnology, Dallas, TX, USA, dilution 1/500); Sialyl-Lewis X (SLe^x^, CSLEX1, DB Pharmingen, San Jose, CA, USA, dilution 1/80); Silayl-Tn (STn, B72.3 [46], dilution 1/5); MUC1 (HMFG1 and HMFG2 [47], both diluted 1/4); Cleaved Caspase-3 (D175, 9661, Cell Signaling Technology, Danvers, MA, USA, dilution 1/800), and Ki67 antibody (ab15580, abcam, Cambridge, United Kingdom, dilution 1/500). The latter two antibodies were used after antigen retrieval with citrate buffer. The lectins used with their working dilutions and specificity were as follows: *Aleuria aurantia* lectin (AAL), dilution 1/3000, Fuc6αGlcNAc; Fucα3GlcNAc; Fucα4GlcNAc; *Sambucus nigra* agglutinin (SNA), dilution 1/3000, Neu5Acα6Galβ4GlcNAc; and *Phaseolus Vulgaris* Leucoagglutinin lectin (L-PHA), dilution 1/2000, Galβ4GlcNAcβ6(Galβ4GlcNAcβ2)Manα6.

### 4.5. Imaging Analysis

The gastric MCTS size, solidity, and roundness features were examined using ImageJ image analysis software with the Fiji image-processing package [48]. First, all images were processed by color deconvolution “H and E” using the green channel, threshold “minimum” was applied, and all objects smaller than 62,000 pixels with and without “include holes” were removed. The resulting mask with “include holes”, which contained a single object, was used to define spheroid size and roundness by ImageJ’s shape descriptor. The more circular an object, the closer its roundness to 1 was. To define solidity, we divided the area of the mask with “include holes” by the area of mask without “include holes”. A completely solid object culminated in a value of 1. All masks were saved and their correctness was confirmed. For ULA plates, pictures of all spheroids were taken, having at least 3 replicates of each condition. For the 3D Petri Dish^®^, each condition consisted of one micromold containing 81 spheroids, and 9 pictures were acquired. 

## Supplementary Materials

The following are available online.
