# Supplementary material for: Multicellular Human Gastric Cancer Spheroids Mimic the Glycosylation Phenotype of Gastric Carcinomas
Source: Molecules. 2018 Oct 30;23(11):2815. doi: 10.3390/molecules23112815 (PMC6278543; doi:10.3390/molecules23112815)
Supplement: Supplementary file 1 [file molecules-23-02815-s001.pdf]

molecules

MDPI

Supplementary Material:

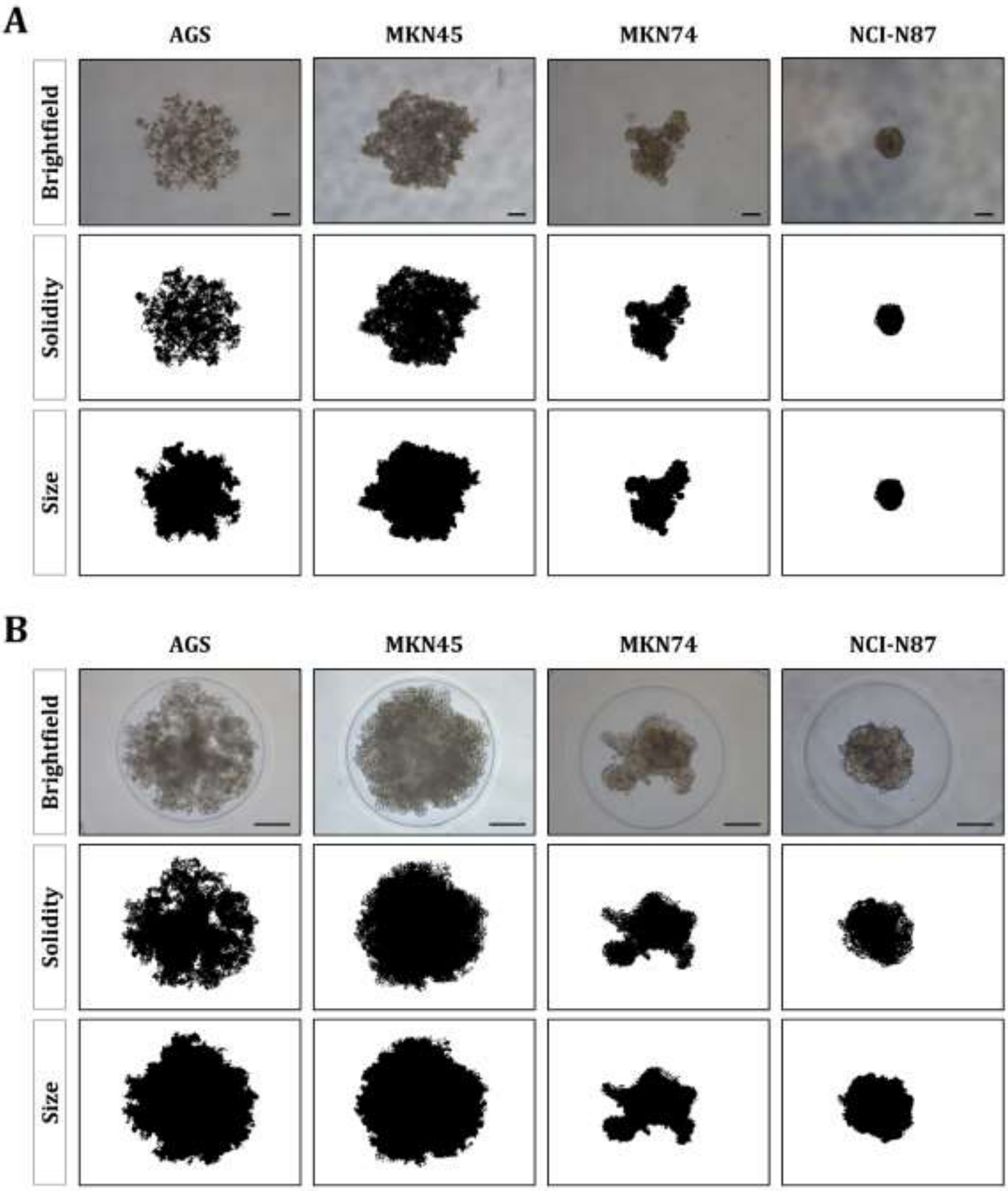

Figure S1. Standardized method for imaging analysis of tumor spheroids. Multicellular tumor spheroids of the four gastric cancer cell lines grown in **(A)** ultralow attachment (ULA) 96-well round-bottomed plates or **(B)** using a 3D Petri Dish® (MICROTISSUES® technology). Images were obtained with a Leica DMi1 microscope and imaging analysis was conducted with open-source Fiji software. Scale bar represents 200 µm.

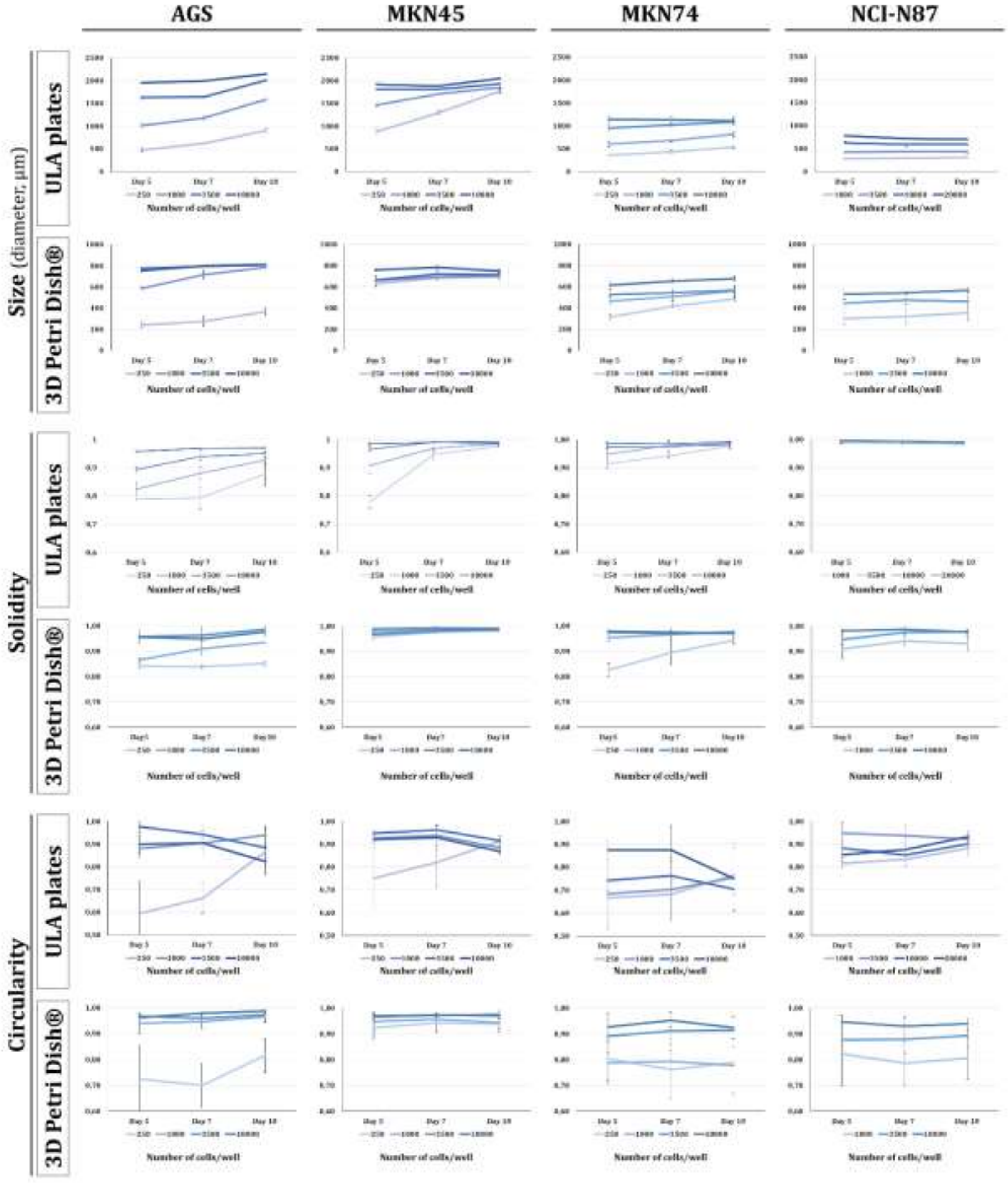

Figure S2. Comparison analysis of two different systems (ULA plates or 3D Petri Dish® technology) to generate suspension cultures of reproducibly sized single spheroids of four gastric cancer cell lines. Automated analysis was applied to characterize size, solidity, and roundness of the multicellular tumor spheroids obtained with the two methodologies. Values are means ± SD of at least n = 3 (ULA) and n = 9 (3D Petri Dish®). For each condition, two independent experiments were conducted.

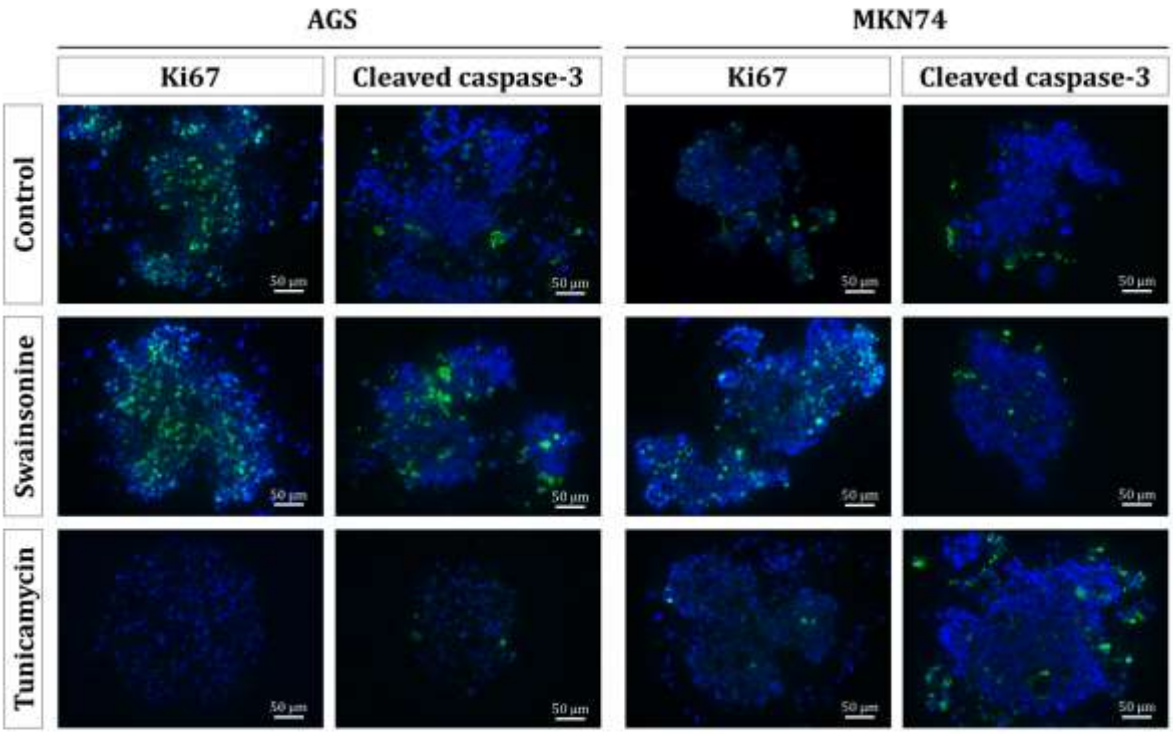

Figure S3. Cell proliferation and apoptotic status of gastric multicellular tumour spheroids (MCTS) treated with *N*-glycan inhibitors. Evaluation of Ki67 and cleaved caspase-3 of AGS, and MKN74 MCTS after treatment with 5 µg/mL of swainsonine or 1 µg/mL of tunicamycin.
